# Diagnosis of insulin autoimmune syndrome using polyethylene glycol precipitation and gel filtration chromatography with *ex vivo* insulin exchange

**DOI:** 10.1111/cen.13179

**Published:** 2016-10-03

**Authors:** David Church, Luís Cardoso, Sonia Bradbury, Catriona Clarke, Anna Stears, Anna Dover, David Halsall, Robert Semple

**Affiliations:** ^1^ The University of Cambridge Metabolic Research LaboratoriesWellcome Trust‐MRC Institute of Metabolic ScienceCambridgeUK; ^2^ The National Institute for Health Research Cambridge Biomedical Research CentreCambridgeUK; ^3^ The Pathology PartnershipDepartment of Clinical Biochemistry and ImmunologyAddenbrooke's HospitalCambridgeUK; ^4^ Department of Endocrinology, Diabetes and MetabolismCentro Hospitalar e Universitário de CoimbraCoimbraPortugal; ^5^ Department of Clinical BiochemistryWestern General HospitalNHS LothianEdinburghUK; ^6^ Cambridge University Hospitals NHS Foundation TrustCambridge Biomedical CampusCambridgeUK; ^7^ Edinburgh Centre for Endocrinology and DiabetesRoyal Infirmary of EdinburghEdinburghUK

## Abstract

**Context:**

Insulin‐binding antibodies may produce severe dysglycaemia in insulin‐naïve patients (‘insulin autoimmune syndrome’ (IAS) or Hirata disease), while rendering routine insulin assays unreliable.

**Objective:**

To assess the performance of clinically used insulin assays and an optimal analytical approach in the context of IAS.

**Design:**

Observational biochemical study of selected patients with hyperinsulinaemic hypoglycaemia.

**Patients:**

Three patients without diabetes with recurrent spontaneous hyperinsulinaemic hypoglycaemia and ‘positive’ insulin antibodies.

**Measurements:**

A panel of clinically used insulin assays (Siemens ADVIA ^®^ Centaur, Siemens Immulite^®^ 2000, DiaSorin LIAISON ^®^ XL, PE AutoDELFIA ^®^ and the Beckman Coulter Access^®^ 2) were used before and after plasma dilution or polyethylene glycol (PEG) precipitation. Anti‐insulin IgG antibodies were measured by Isletest^™^‐IAA ELISA. Gel filtration chromatography (GFC) was undertaken with and without preincubation of plasma with exogenous insulin.

**Results:**

Dilution of IAS plasma with assay‐specific buffer increased insulin recovery, supporting negative immunoassay interference by antibodies. PEG precipitation of IAS plasma decreased insulin recovery using all assays except the Immulite^®^ 2000. GFC discriminated high molecular weight and monomeric insulin, while *ex vivo* addition of exogenous insulin to plasma increased insulin bound to antibody, thereby improving the sensitivity of detection of insulin immunocomplexes.

**Conclusions:**

Immunoprecipitation with PEG must be used with caution in screening for insulin–antibody complexes as results are assay dependent. GFC with addition of exogenous insulin can identify significant insulin immunocomplexes with enhanced sensitivity, with attendant greater clinical utility and avoidance of radiolabelled reagents.

## Introduction

The existence of hormone–immunoglobulin complexes (so called ‘macrohormones’) is well known. Such complexes pose a significant challenge to the measurement of hormones by immunoassay and may also interfere with bioactivity of the hormones sufficiently to cause clinical disorders. Macroprolactin is the best characterized macrohormone.^1^ However, macrocomplexes have also been described for many other hormones including luteinising hormone,^2^ follicular‐stimulating hormone,^3^ thyroid‐stimulating hormone,^4^ human chorionic gonadotrophin^5^ and also insulin^6^. As a result of insulin having a short plasma half‐life, and because either excess insulin action or deficient insulin action may lead to dysglycaemia and death, over minutes and hours, respectively, anti‐insulin antibodies are potentially particularly hazardous to health.

Demonstration of insulin‐binding immunoglobulin was first reported in the circulation of patients treated with exogenous insulin in 1955,^7^ and such antibodies were the focus of many studies when animal‐derived insulins were commonly used. Some such insulin‐binding antibodies in plasma have been shown to alter insulin pharmacokinetics and/or pharmacodynamics, both in patients naïve to insulin therapy (‘insulin autoimmune syndrome’ (IAS) or ‘Hirata disease’)^8^ and in patients with labile diabetes treated with modern genetically engineered insulin analogues.^9^ In both situations, patients may present with insulin resistance and/or hypoglycaemia, as the antibody serves both to bind and sequester acutely released/administered insulin, and as a source of long‐acting bioavailable insulin as insulin dissociates from complexes in the fasting state.^10^

Anti‐insulin antibody assays are now widely available commercially, and positive results are returned in a significant number of patients treated with insulin, and in some insulin‐naïve control subjects.^11^ These assays thus have low specificity for detection of patients with antibodies that derange insulin kinetics to a clinically significant degree. Several adjunctive approaches have consequently been used in the assessment of anti‐insulin antibodies, most commonly including immunoprecipitation with polyethylene glycol (PEG), a common tool in the evaluation of ‘macro‐analytes’.^12^ Nevertheless, formal assessment of this technique in tandem with modern clinical insulin immunoassays has not been published, which is important as PEG immunoprecipitation may compromise performance of some immunoassays.

Gel filtration chromatography (GFC) is often cited as the gold standard method for detecting macro‐analyte complexes and has been used to demonstrate the presence of high molecular weight (HMW) insulin immunoreactivity in patients with dysglycaemia.^13^ However, GFC‐based approaches are limited by the dilution of the sample that occurs during filtration, meaning that the analyte must be present at sufficiently high concentration to be above the assay detection limit postfiltration. A further concern is that dilution may disturb the equilibrium established between free and bound hormone present *in vivo*. Detection of macro‐analyte complexes may also be confounded by the so called ‘heterophilic’ antibody interference, caused by endogenous antibodies that cross‐react with the immunoassay components rather than the analyte in question; both false‐negative and false‐positive interferences are possible because assay components can be either blocked or cross‐linked in the absence of analyte.^14^, ^15^ Both types of interferences are assay dependent, so method comparison studies alone may neither detect nor distinguish between them. While the distinction between macro‐analyte and heterophilic antibody interference may appear semantic, it is important for clinical decision‐making, particularly in the case of insulin analysis, where macrohormone complexes may affect insulin kinetics *in vivo* and cause life‐threatening metabolic complications, whereas heterophile interference is purely an analytical challenge.

In this report, the performance of different commercially available insulin assays in the context of dilution and PEG precipitation studies is assessed, and a protocol for detecting macroinsulin complexes using GFC, with incorporation of *ex vivo* assessment of increase/exchangeability of insulin binding to improve sensitivity, is described.

## Materials and methods

### Patients studied and sample collection

Three patients without diabetes were evaluated by the UK Severe Insulin Resistance Supraregional Assay Service, Addenbrooke's Hospital, Cambridge. Blood samples were collected on wet ice, and plasma/serum were rapidly separated and frozen at −80 °C until analysis. Surplus plasma from patient 1 was used for the assay comparison study. All experimental procedures were performed in accordance with the World Medical Association Declaration of Helsinki (2000).

### Anti‐insulin antibody measurement

Serum anti‐insulin IgG was measured using the Biomerica Isletest^™^‐IAA ELISA (Biomerica, Irvine, CA, USA) semi‐quantitative method.

### Insulin immunoassays

Insulin was measured in pooled plasma from patient 1 using a panel of commercially available platforms, namely Siemens ADVIA^®^ Centaur (Siemens Healthcare Diagnostics, Camberley, Surrey, UK), Siemens Immulite^®^ 2000 (Siemens Healthcare Diagnostics), DiaSorin LIAISON^®^ XL (DiaSorin, Dartford, Kent, UK), PerkinElmer AutoDELFIA^®^ (Wallac Oy, Turku, Finland) and the Beckman Coulter Access^®^ 2 (Beckman Coulter, High Wycombe, Buckinghamshire, UK). Insulin analysis was performed in singleton, based on known assay performance characteristics and in line with routine diagnostic laboratory practice. Venous plasma insulin (LIAISON^®^ XL), C‐peptide (LIAISON^®^ XL) and glucose (Siemens ADVIA^®^ 2400 Chemistry System) were also measured in nonfasting plasma for all three patients.

### Dilution studies

Insulin was measured in neat plasma and then following a 1:4 dilution with assay‐specific diluent, with starting insulin concentrations calculated using the dilution factor where required. Surplus pooled anti‐insulin antibody‐negative plasma from exogenous insulin‐naïve patients was used as a control.

### Immunoprecipitation with polyethylene glycol

The same sample from patient 1 analysed in the dilution studies above was used. A 25% w/v solution of BDH Prolabo^®^ PEG 6000 (VWR International, Lutterworth, Leicestershire, UK) was prepared using deionized water; 0·9% saline was prepared using BDH Prolabo® 18% w/v sodium chloride solution (VWR International) and deionized water. The pooled sample from patient 1 was diluted 1:1 with 25% w/v PEG and mixed for 10 s using a vortex and then centrifuged at 13 200 ***g*** for 15 min. Insulin concentration was measured in the neat supernatant using the panel of insulin immunoassays. To overcome sampling error with the LIAISON^®^ XL (likely exacerbated by the increased sample viscosity due to PEG), a 1:1 dilution of the PEG supernatant was also analysed for insulin. Control plasma was as above.

Insulin recovery was then determined in the same way for all the three patients in nonfasting plasma and ten control samples. Analysis was performed using the LIAISON^®^ XL, because it demonstrates specificity for human insulin^16^ and has the convenience of random access analysis.

### Gel filtration chromatography

Five hundred microlitres of plasma was loaded onto a HiLoad 16/60 Superdex 75 (120 ml) size‐exclusion column in combination with a 25 mmol/l Tris/0·52 mol/l NaCl buffer mobile phase at pH 7·4, with a flow rate of 1 ml/min. Optimization studies demonstrated that the addition of bovine serum albumin (BSA) to fraction collector tubes before GFC improved insulin recovery from the column, achieving >70%. Six millilitre elution volume fractions with 1 ml bovine serum albumin (BSA) (final volume 7 ml, calculated BSA concentration 40 g/l) were collected in Cellstar^®^ polypropylene tubes (Greiner Bio‐One, Stonehouse, Gloucestershire, UK). A total of 36–114 ml eluted volume was collected.

GFC was performed using the ÄKTAprime plus^™^ liquid chromatography system (GE Healthcare, Uppsala, Sweden), and ultraviolet (UV) absorbance at 280 nm was recorded. The chromatography method demonstrated good precision with elution volume coefficient of variation of 6% for immunoglobulin (*n* = 30; mean elution volume 49 ml).

Samples were analysed for insulin using the LIAISON^®^ XL immunoassay. This assay was chosen because in‐house data supported high analytical sensitivity (1·2 pmol/l) and acceptable coefficient of variation at lower insulin concentrations (8·6% at 34 pmol/l; *n* = 244).

### Insulin exchange studies

A total of 990 μl of neat plasma was mixed with 10 μl of insulin/insulin analogue of the desired concentration. The samples were incubated on a rotator at 21 °C for 24 h before being run through the GFC protocol described above in parallel with samples before exogenous insulin addition.

## Results

The three patients studied were female, presented with recurrent spontaneous hyperinsulinaemic hypoglycaemia (glucose level below 2·5 mmol/l confirmed on laboratory evaluation of venous blood), ‘positive’ insulin antibodies and were not treated for diabetes mellitus at the time of blood sampling. Nonfasting samples were analysed for insulin, C‐peptide and glucose (Table 1). All the three patients had detectable plasma C‐peptide and a high insulin:C‐peptide ratio^17^ at times of euglycaemia. Subsequent investigation aimed to establish whether these antibodies explained the presenting metabolic disorder.

**Table 1:** Demographic characteristics and initial biochemical profile of patients studied

Patient number	Sex	Age (years)	Nonfasting plasma glucose (mmol/l)	Insulin (pmol/l)	Insulin recovery following PEG precipitation (%)	C‐peptide (pmol/l)	Prior clinical diagnoses
1	Female	56	4·3	7020	8	3297	Autoimmune hypothyroidism
2	Female	28	7·7	1650	63	3240	Autoimmune hypothyroidism; alcoholic hepatic cirrhosis; systemic lupus erythematosus
3	Female	37	8·0	69 000	4	4960	Antiphospholipid syndrome

### Dilution studies

**Figure 1:**
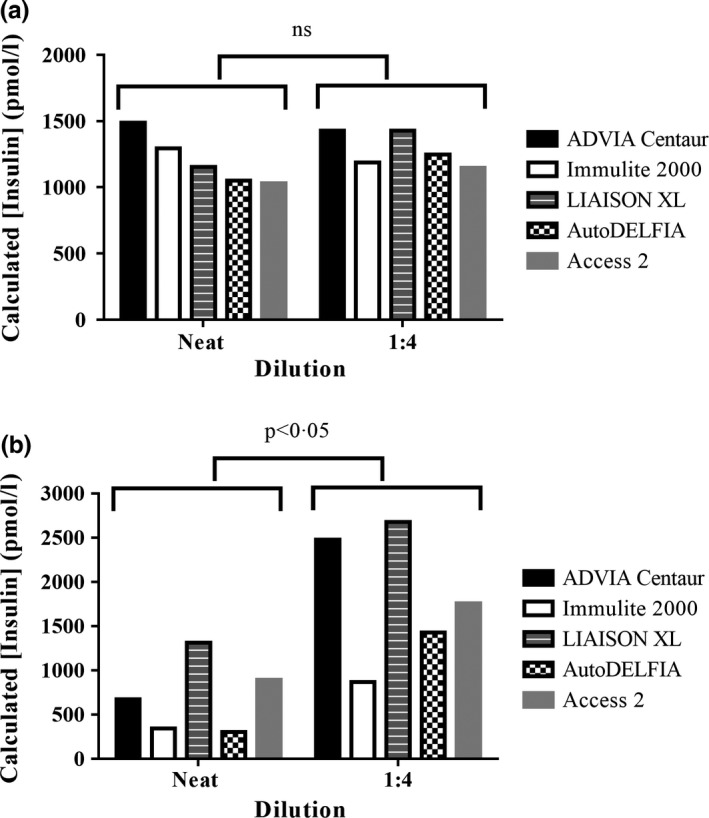
Effect of plasma dilution and anti‐insulin antibodies on insulin determination by a panel of insulin immunoassays. Calculated insulin concentration plotted against plasma dilution for antibody‐negative control plasma (a) and IAS plasma from patient 1 (b). Insulin measurements were made using a panel of assays (Siemens ADVIA ^®^ Centaur, Siemens Immulite^®^ 2000, DiaSorin LIAISON ^®^ XL, PE AutoDELFIA ^®^ and the Beckman Coulter Access^®^ 2) as indicated. Neat control plasma concentrations and corresponding calculated starting concentrations derived from assay of diluted samples were compared using the Mann–Whitney *U*‐test.

To assess insulin assay linearity in a plasma sample with insulin‐binding antibodies, dilution of the pooled heparinised plasma from patient 1 was undertaken. As the purpose of this experiment was to observe the effects of dilution on native plasma, a sample was chosen with an insulin concentration predetermined to be within the dynamic range of the insulin assays used. This removed the requirement to predilute the sample, which could affect the insulin–immunoglobulin binding equilibrium, or cause an assay‐dependent matrix effect. Pooled samples from patients without measurable insulin antibodies were used as a control. Five different diagnostic immunoassays were studied. Agreement among insulin assays was consistent with known method bias.^18^, ^19^ Samples were then reanalysed following a 1:4 dilution with assay‐specific diluent. All assays showed linear insulin recovery in the control sample (Fig. 1a); however, there was increased recovery of insulin (Mann–Whitney test *P* < 0·05) using all five assays in the IAS plasma following dilution (Fig. 1b).

### Immunoprecipitation with polyethylene glycol

**Figure 2:**
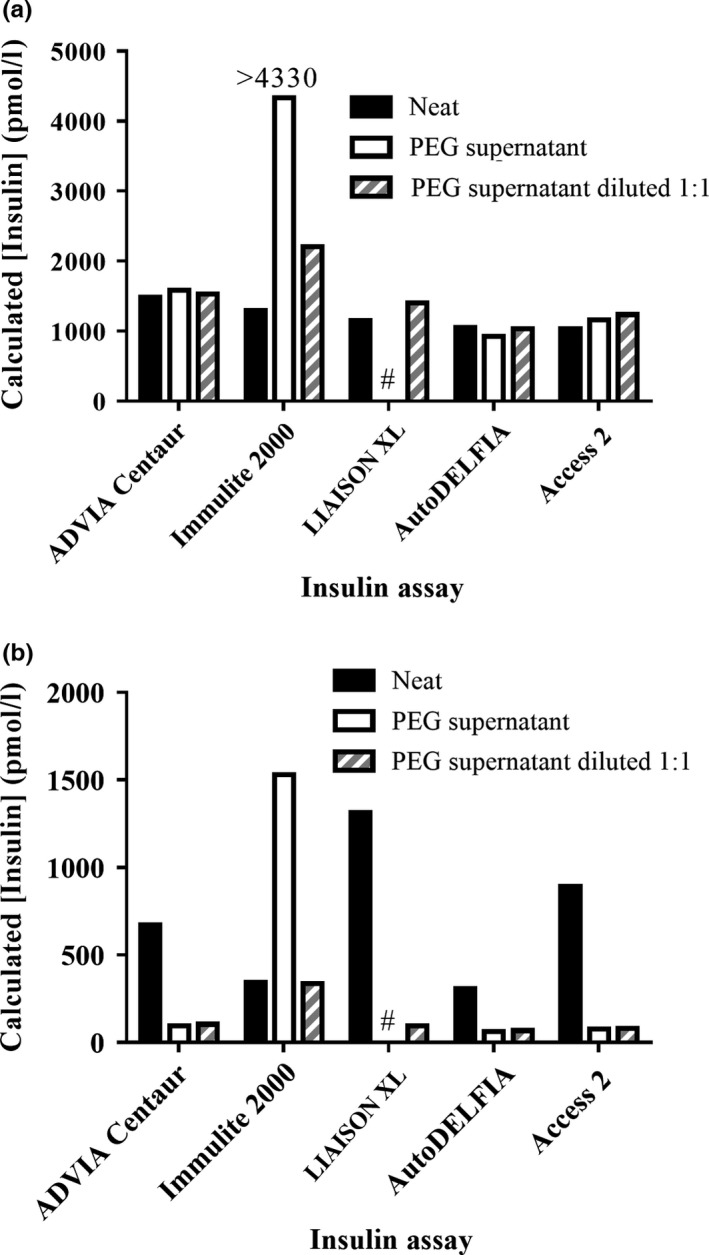
Effect of PEG precipitation and anti‐insulin antibodies on insulin determination by a panel of insulin immunoassays. Calculated insulin concentration in neat plasma, PEG supernatant and PEG supernatant following 1:1 dilution in assay buffer for antibody‐negative control plasma (a) and IAS plasma from patient 1 (b) is shown. Insulin measurements were made using a panel of assays (Siemens ADVIA ^®^ Centaur, Siemens Immulite^®^ 2000, DiaSorin LIAISON ^®^ XL, PE AutoDELFIA ^®^ and the Beckman Coulter Access^®^ 2) as indicated. The LIAISON ^®^ XL analysed was unable to analyse the PEG supernatant and reported a sample error (#).

To identify whether PEG can be used to screen for the presence of insulin‐binding antibodies using the different insulin assays, insulin recovery in supernatant following PEG precipitation of plasma was then studied. The same neat plasma samples analysed in the dilution studies above were diluted 1:1 with 25% w/v PEG and, following centrifugation, insulin was measured in the supernatant. Insulin determinations using three of the five assay platforms of the control sample demonstrated the expected 50% dilutional effect with PEG. The exceptions were the Immulite^®^ 2000 assay, which demonstrated paradoxically increased insulin immunoreactivity following dilution with PEG (Fig. 2a), and the LIAISON^®^ XL assay, which repeatedly reported a sample error. To reduce the viscosity of the supernatant, a 1:1 dilution with assay‐specific diluent was performed. Linearity was demonstrated in four of the five assays with respect to neat plasma, including the LIAISON^®^ XL assay. The Immulite^®^ 2000 still yielded a higher calculated result than measured in neat plasma, although the over‐recovery was less than seen for the 1:1 PEG diluted sample. Subsequently, a lower reference limit of 102% was defined for the LIAISON^®^ XL assay by studying 10 further control plasma samples (median 107%; 95% confidence interval 102–112%).

Using plasma from patient 1, three of the five assays demonstrated lower insulin recoveries in PEG supernatant; however, the Immulite^®^ 2000 assay, as before, demonstrated an increased insulin immunoreactivity following dilution with PEG (Fig. 2b) and the LIAISON^®^ XL, as before, reported a sample error. Further assay of 1:1 diluted PEG supernatant using the ADVIA^®^ Centaur, AutoDELFIA^®^ and the Coulter Access^®^ 2 (which had recorded results in the supernatant) demonstrated linearity with respect to the PEG supernatant itself, but not to the neat plasma. The Immulite^®^ 2000 assay yielded a much lower calculated insulin concentration than expected in the 1:1 diluted PEG supernatant when compared to the PEG supernatant. The calculated concentration in the diluted PEG supernatant corresponded exactly to the value measured in the neat plasma; however, the studies above in the control plasma demonstrate that this is likely to be coincidental and potentially misleading in a clinical diagnostic context, given the over‐recovery of insulin in this assay in the presence of PEG.

### GFC with insulin exchange studies

**Figure 3:**
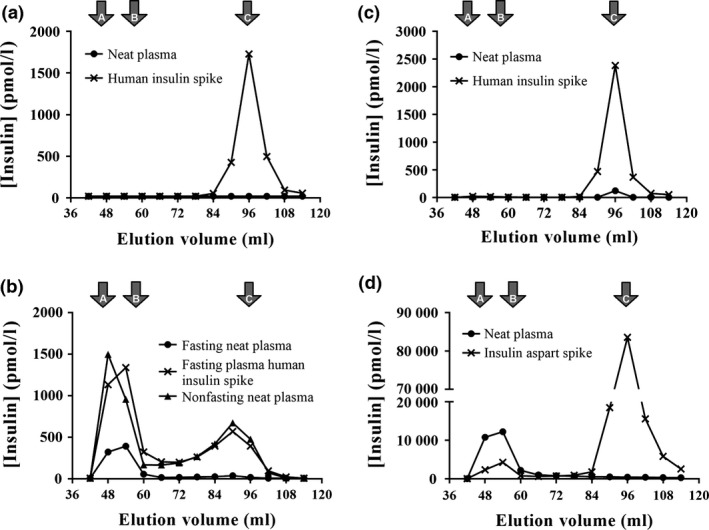
Demonstration of reversible insulin binding to immunocomplexes using gel filtration chromatography of plasma. Results of insulin assay after gel filtration chromatography of insulin antibody‐negative control plasma or patient plasma are shown: (a) insulin antibody‐negative control plasma pre‐ and posthuman insulin spike, (b) patient 1 nonfasting plasma or fasting plasma pre‐ and posthuman insulin spike; (c) patient 2 nonfasting plasma pre‐ and posthuman insulin spike; (d) patient 3 nonfasting plasma pre‐ and postinsulin aspart spike. Elution volumes of immunoglobulin (A), albumin (B) and monomeric insulin (C) are shown. Insulin concentrations were measured using the DiaSorin LIAISON ^®^ XL.

Analysis was extended to the use of GFC to seek direct evidence for the presence of macroinsulin complexes. In a control sample with a measured plasma insulin concentration of 14pmol/l, no insulin peaks could be seen in the eluate (Fig. 3a); this is expected due to the dilution incurred during filtration resulting in insulin concentrations in the eluted fractions beyond the lower limit of the insulin assay. When the sample was preincubated with exogenous human insulin to increase the measured insulin concentration to 7655 pmol/l, the exogenous insulin was eluted in fractions consistent with the monomeric insulin. The combination of GFC with insulin preincubation was then tested on the plasma from the three patients.

A nonfasting sample from patient 1 with an estimated insulin concentration of 7480 pmol/l showed peaks of immunoreactivity consistent with both monomeric and a HMW species (macroinsulin). A fasting sample with an estimated insulin level of only 774 pmol/l in neat plasma from patient 1 was next used, to challenge the discriminatory power of GFC at lower insulin concentrations. Despite the lower insulin concentration, a macroinsulin peak was still discernible (Fig. 3b). The same sample was then preincubated, as before, with exogenous insulin, increasing measured insulin concentration to 7840 pmol/l. The macroinsulin peak was greatly accentuated, consistent with an excess insulin‐binding capacity by the insulin‐binding antibody. The exogenous insulin addition not only increases the sensitivity of the GFC method for detecting insulin‐binding antibodies, but also demonstrates that the insulin‐binding complex is in dynamic equilibrium with free insulin.

Undiluted plasma from patient 2, with an insulin concentration of 198 pmol/l, failed to demonstrate a macroinsulin peak following GFC (Fig. 3c). Moreover, incubation with human insulin to increase the measured concentration to 8720 pmol/l, although enormously increasing the monomeric insulin peak, failed to unmask any HMW insulin peak. This result is consistent with the immunoassay‐detected antibody not having sufficient concentration and/or affinity to form demonstrable insulin complexes *ex vivo* using this method. It is therefore unlikely that significant reservoirs of bound insulin will accumulate *in vivo*, so any clinical sequelae associated with the positive anti‐insulin antibody are unlikely.

Patient 3 posed a different challenge, with a plasma insulin concentration recorded as 69 000 pmol/l. GFC of neat plasma demonstrated a predominance of HMW, rather than monomeric, insulin immunoreactivity (Fig. 3d). This would be consistent with the presence of an antibody with very high insulin‐binding capacity; however, the presence of heterophilic antibody interference is an alternative explanation. As heterophilic antibodies bind immunoassay reagents rather than insulin *per se,* a demonstration of insulin exchange into the HMW fraction would exclude heterophile interference. Thus, rather than attempting to increase the HMW insulin fraction further with exogenous human insulin, we aimed to demonstrate exchangeability by assessing the ability of exogenous analogue insulin to exchange into the macroinsulin fraction. Insulin aspart was chosen as it has been previously demonstrated to show low cross‐reactivity with the LIAISON^®^ XL assay.^16^ Analysing GFC fractions using the LIAISON^®^ XL assay showed an expected decrease in the macroinsulin fraction consistent with displacement of native insulin by aspart. The monomeric insulin fraction increased due to the displaced native insulin and due to the (low) cross‐reactivity with large amounts of unbound insulin aspart. Subsequent analysis of GFC fractions with and without insulin aspart using an assay with higher cross‐reactivity with insulin aspart^16^ confirmed native insulin could be displaced from the macroinsulin complex using insulin aspart binding by antibody (See Fig. S1).

## Discussion

Immunocomplexing of hormones by endogenous antibodies to produce so called ‘macrohormones’ can lead to apparent elevation, sometimes dramatic, of measured blood concentrations, but usually without the expected physiological responses as the hormone:immunoglobulin complexes are presumed to be biologically inactive. Detection of such complexes can explain aberrant endocrine results and has great diagnostic utility.^1^, ^20^, ^21^ Unlike most macrohormone complexes which appear biologically inactive, autoantibodies with the ability to accumulate insulin *in vivo* pose a major physiological risk as well as an analytical challenge: insulin autoantibodies may not only attenuate the acute hypoglycaemic action of insulin through sequestration of free insulin, but also commonly cause postabsorptive hypoglycaemia as insulin dissociates from the complex at physiologically inappropriate times. To have these effects, insulin autoantibodies must have relatively high concentration and an affinity that allows for insulin dissociation within a physiologically relevant time‐scale. Simply detecting the presence of the antibodies by immunoassay is not informative on these critical points. This motivated us to explore in further detail laboratory methods for identifying clinically significant insulin autoantibodies in the context of widely available immunoassays.

In the presence of anti‐insulin antibodies, the insulin concentration determined by immunoassay of plasma will not necessarily reflect either the total insulin (antibody‐bound plus free) or free insulin, as the equilibrium between bound and free insulin may be affected by diluting the sample in assay buffer. This is likely to be assay dependent as different buffers, dilutions and capture antibodies may be used, and it underlies the need for more detailed biochemical assessment in such cases. Nonlinear immunoassay results with dilution of plasma, caused by dissociation of immunocomplexes, are widely used as a specific but insensitive indicator of immunoassay interference, with such nonlinearity depending upon the affinity of the assay antibodies relative to that of the insulin autoantibody if the epitope is shared. Assay incubation time may affect results if there is insufficient time for equilibrium to be re‐established between the immunoassay reagents and the putative hormone:immunoglobulin complex. In our study, concordance among a panel of widely used commercial assays was considerably reduced in the presence of insulin autoantibodies, highlighting a method‐dependent sensitivity to this type of interference. However, all of the assays used did show nonlinearity (increased insulin recovery) with dilution. These observations confirm the value of dilution studies in this context, but the measurement is indirect, and the sensitivity of this method remains unproven.

Immunoprecipitation with PEG is another common screening tool for insulin–antibody complexes; however, this technique is also assay dependent. We found PEG precipitation of plasma from a patient with insulin autoantibodies demonstrated decreased insulin recovery in PEG supernatant using all assays except the Immulite^®^ 2000, which may relate to a matrix effect caused by the viscosity of PEG in this assay. PEG precipitation is known also to be susceptible to sample‐specific matrix effects,^12^ to exhibit differential precipitation of some immunoglobulin subclasses, notably IgA,^22^ and is dependent on the ability to measure insulin accurately if the original plasma insulin concentration is low. It follows that, if the limitations of this technique are considered, PEG precipitation can be used as a simple practical method to screen for the presence of insulin–antibody complexes in plasma.

We have now developed a GFC method coupled to *ex vivo* insulin binding/exchange to refine identification of anti‐insulin autoantibodies capable of aggregating insulin reversibly without recourse to radiolabelled materials. As expected, macroinsulin was easily detectable by GFC in a patient with very high measured plasma insulin levels and multiple lines of biochemical and clinical evidence supported the autoantibodies to be clinically significant. The sensitivity of GFC is limited in some cases, however, by low‐input insulin concentration, for example when blood is drawn after a prolonged fast, and potentially also by the dissociation of insulin from weakly bound complexes during sample dilution during the filtration process. Using a sample from a patient with relatively low insulin concentration, *ex vivo* incubation with a high concentration of free insulin increased sensitivity of the GFC method by pushing the binding equilibrium in favour of insulin binding, permitting unequivocal demonstration of the ability of the plasma to immunocomplex insulin. We note also that this approach has potential utility also in cases of sera stored at room temperature, where insulin may have been degraded but where anti‐insulin antibodies persist.

Demonstration of insulin exchange had additional value to refute the presence of heterophile antibodies as the explanation for the high molecular weight peak of insulin immunoreactivity. Increased sensitivity of the GFC technique also enhances the negative predictive value of the test, as in the second patient we describe, in whom advanced liver disease offered an alternative explanation for fasting hypoglycaemia, rather than insulin sequestration by an insulin autoantibody.

In a development of this approach, we report that *ex vivo* use of analogue insulins and analogue‐specific insulin assays in eluates further increases the clinical utility. Using these to demonstrate the ability of analogue insulin to exchange with native insulin binding to immunocomplexes, as in the third patient described, who had an extremely high macroinsulin detected on GFC, effectively ruled out heterophilic antibodies as the explanation for abnormal immunoassay results and the high molecular weight immunoreactivity fraction on GFC. This method is considerably more convenient than exchange studies using radiolabelled insulin.^23^

It is well understood that many immunoassay‐detected anti‐insulin antibodies are of little or no clinical significance,^24^ and so, there is a pressing need to develop robust laboratory approaches to stratify such antibodies and to identify those likely to perturb insulin pharmacokinetics and pharmacodynamics. We here extend previous reports of the use of dilution studies, PEG precipitation and GFC to delineate the strengths and limitations of the approaches in the context of widely used commercial immunoassays. While each approach has utility in the laboratory investigation of anti‐insulin autoantibodies, full understanding of the performance of these approaches, and development of efficient diagnostic algorithms, will require their application to a larger population of patients with dysglycaemia and detectable anti‐insulin antibodies.

## Funding

D.C. is funded by a Diabetes Research & Wellness Foundation Sutherland‐Earl Clinical Fellowship (RG68554) and R.S. by the Wellcome Trust (Grant WT098498). Funding was also received from the Medical Research Council (MRC_MC_UU_12012/5), the United Kingdom National Institute for Health Research (NIHR), Cambridge Biomedical Research Centre.

## Conflict of interest

The authors have declared that no conflict of interest exists.

## Supporting information


**Fig. S1**. Demonstration of insulin aspart binding to immunocomplexes using gel filtration chromatography of plasma.
