# Supplementary figures and images for: Diagnosis of insulin autoimmune syndrome using polyethylene glycol precipitation and gel filtration chromatography with ex vivo insulin exchange
Source: Clin Endocrinol (Oxf). 2016 Oct 3;86(3):347–53. doi: 10.1111/cen.13179 (PMC5324546; doi:10.1111/cen.13179)

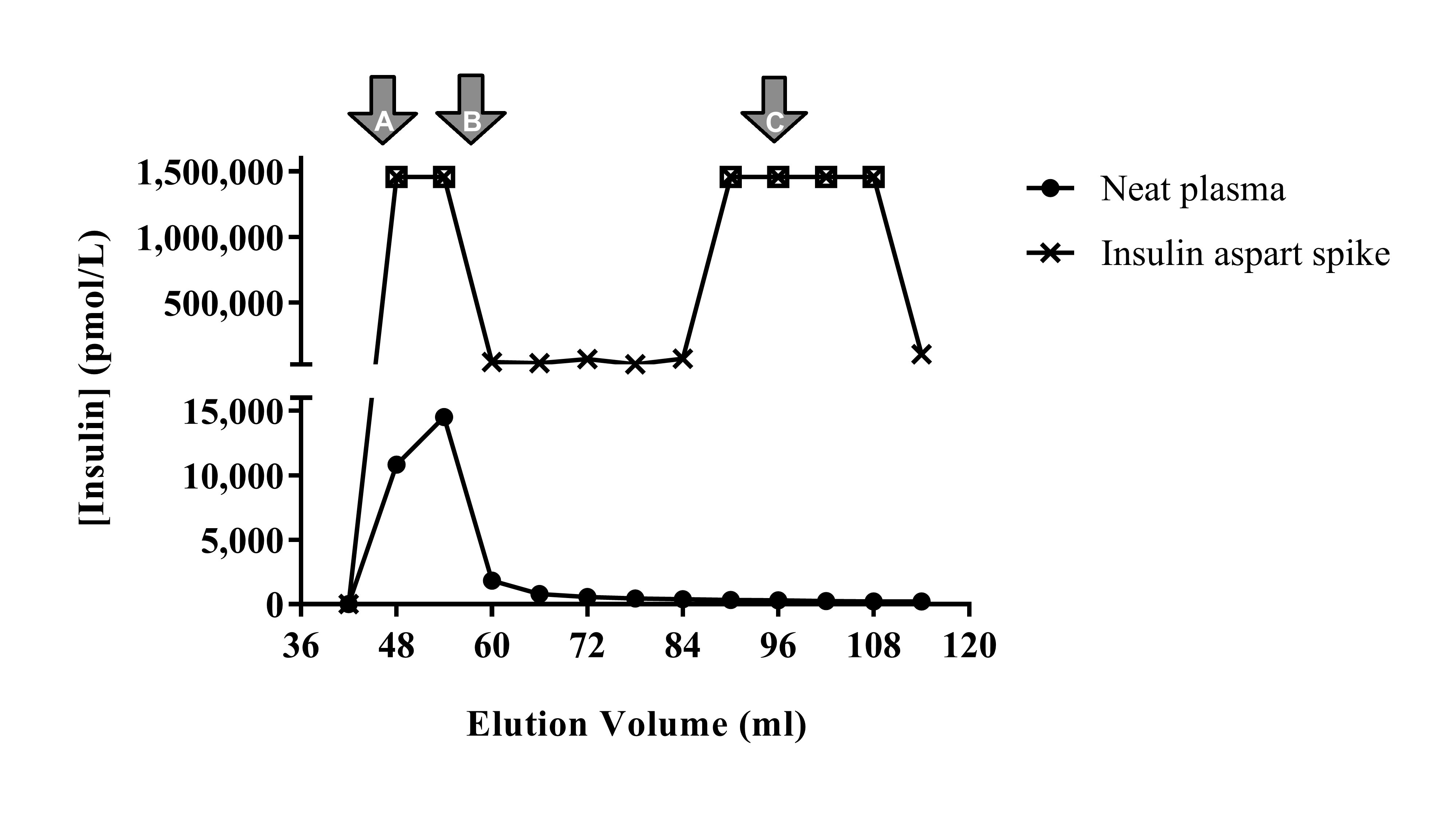

Supplement: Supplementary file 1 — Fig. S1. Demonstration of insulin aspart binding to immunocomplexes using gel filtration chromatography of plasma. [file CEN-86-347-s001.tif]
