# Supplementary material for: Diagnosis of insulin autoimmune syndrome using polyethylene glycol precipitation and gel filtration chromatography with ex vivo insulin exchange
Source: Clin Endocrinol (Oxf). 2016 Oct 3;86(3):347–53. doi: 10.1111/cen.13179 (PMC5324546; doi:10.1111/cen.13179)
Supplement: Supplementary file 2 [file CEN-86-347-s002.docx]

**Supplementary figure: Demonstration of insulin aspart binding to immunocomplexes using gel filtration chromatography of plasma.** Patient 3 non-fasting plasma pre- and post *ex vivo* insulin aspart incubation. Elution volumes of immunoglobulin (A), albumin (B) and monomeric insulin (C) are shown. Insulin concentrations were measured using the Beckman Coulter Access^®^ 2 assay. Results were truncated at 1,500,000pmol/L (truncated data points indicated by □).
